# Tuberculosis control in people living with HIV/AIDS**^1^**

**DOI:** 10.1590/1518-8345.1187.2798

**Published:** 2016-09-09

**Authors:** Gabriela Tavares Magnabosco, Lívia Maria Lopes, Rubia Laine de Paula Andrade, Maria Eugênia Firmino Brunello, Aline Aparecida Monroe, Tereza Cristina Scatena Villa

**Affiliations:** 2 PhD, Post-doctoral fellow, Escola de Enfermagem de Ribeirão Preto, Universidade de São Paulo, PAHO/WHO Collaborating Centre for Nursing Research Development, Ribeirão Preto, SP, Brazil.; 3 MSc, Doctoral student, Escola de Enfermagem de Ribeirão Preto, Universidade de São Paulo, PAHO/WHO Collaborating Centre for Nursing Research Development, Ribeirão Preto, SP, Brazil.; 4 PhD, RN, Escola de Enfermagem de Ribeirão Preto, Universidade de São Paulo, PAHO/WHO Collaborating Centre for Nursing Research Development, Ribeirão Preto, SP, Brazil.; 5 PhD, Professor, Escola de Enfermagem de Ribeirão Preto, Universidade de São Paulo, PAHO/WHO Collaborating Centre for Nursing Research Development, Ribeirão Preto, SP, Brazil.; 6 PhD, Professor, Escola de Enfermagem de Ribeirão Preto, Universidade de São Paulo, PAHO/WHO Collaborating Centre for Nursing Research Development, Ribeirão Preto, SP, Brazil.; 7 PhD, Full Professor, Escola de Enfermagem de Ribeirão Preto, Universidade de São Paulo, PAHO/WHO Collaborating Centre for Nursing Research Development, Ribeirão Preto, SP, Brazil.

**Keywords:** Acquired Immunodeficiency Syndrome, Tuberculosis, Chronic Disease, Health Systems

## Abstract

**Objective:**

to analyze the offering of health actions and services for the control of tuberculosis for people living with HIV/AIDS being followed up in the Specialized Care Services for HIV/AIDS in Ribeirão Preto, SP, Brazil.

**Method:**

quantitative, exploratory survey study. Participated 253 people living with HIV/AIDS followed up by this service, considering as inclusion criteria: individuals older than 18 years living in the city and not inmates. Data collection was conducted from January 2012 to May 2013 through interviews with the support of a specific instrument. Data were analyzed using indicators and a composite index.

**Results:**

the offering of services for the control of tuberculosis in people living with HIV/AIDS by municipal services was considered as intermediate, reinforcing the need for better planning for comprehensive assistance, coordination of professionals in teams and among the services network, in addition to professional training and continuing education.

**Conclusion:**

it is necessary to implement strategies that promote shared actions between TB and HIV / AIDS programs and between different services in order to strengthen the local care network, aimed at producing an individualized care, comprehensive and responsive.

## Introduction 

Although Tuberculosis (TB) is a disease whose cure has been possible for decades now, it is still recognized as a current and persistent public health problem. Linked to poverty, poor income distribution and poor quality of living conditions of the population^1^ ^-^ ^3^, TB leads to the death of about six thousand people a year in Brazil, and is aggravated by the increased numbers of cases of Acquired immunodeficiency Syndrome (AIDS)^4^. It is considered that the HIV infection is one of the most important known risk factors for tuberculosis^5^ ^-^ ^6^, so that people living with HIV/AIDS (PLWH) are 21 to 34 times more likely to develop active TB as compared to the general population^7^.

In Brazil, the active TB in PLWH is the condition of greater impact on mortality from AIDS and TB. In 2011, among the cases of TB reported in the SINAN (71,000), about 10% had TB / HIV, and 6% of the mortality rate was related to the overlap of both infections^8^.

In this sense, both diseases are major challenges^9^ for health services and government agencies^10^ ^-^ ^11^. It is recognized the need for effective integration of the actions that are offered both within the service itself, by the teams of TB control programs (TCP) and by the ones of HIV / AIDS, and among the different health care points for creating an effective network of coherent services, with appropriate assistance to TB / HIV co-infection.

In this way, to think in the care services for PLWH with a focus on TB control, implies the establishment of a plan of care that is proactive, integrated and continuous^12^. It is understood that the assistance dynamics both for care to PLWH as well as for TB, must transcend the mere apprehension of epidemiological rates, assuming interdisciplinary practices that take into account the relationships and living conditions as they exist in a given territory in social vulnerability, enabling better planning and reorganization of health technologies ^(11.13)^.

It is noteworthy that the studies analyzed in a survey of the literature on the subject in the last five years, pointed to the existence of gaps in the production of scientific knowledge in relation to the incorporation of aspects of the context of the subjects and the imbrication of this context with the care process. In this regard, it is remarked the importance of the development of this operational study, which allowed the identification of actions and health services taking into account the complexity of the management of these disorders.

Understanding that TB is a leading cause of death among PLWH, it should be imperative a greater commitment to prevention and diagnosis of this population, articulation and coordination between professionals, actions and health services. In this sense, the study aimed to analyze the supply of health actions and services for the control of TB in PLWH in Ribeirão Preto-SP.

## Method

Descriptive, exploratory survey study using a quantitative approach, performed in Ribeirão Preto. Ribeirao Preto's estimated population in 2011 was 614,759 inhabitants^14^. That same year, the public health care in the municipality was organized in five health districts, with five emergency units, 76 primary care services (so-called "Traditional units", Care Units Primary Care-AB and Health of the Family Units-HFU), five units of secondary care (District Basic Units-DBU), in which the five Specialized Care Services (SCS) are located, and where the sexually transmitted disease program/AIDS is executed, and nine tertiary services.

The clinical and therapeutic monitoring of cases of HIV/AIDS diagnosed and reported in the city was carried out by specialized teams of SCS, designated with the letters "A", "B", "C", "D" and "E". Also noteworthy is the fact that the SCSs shared the same physical structure of the TCP in each of the health districts, and the same professionals composed health teams of both programs at the time of the study.

It is important to note that the monitoring of PLWH infected with Mycobacterium tuberculosis, for both latent infection and for active TB, is the responsibility of the teams of SCS. However, the Ministry of Health (MOH) recommends that the management of both infections should be conducted in a coordinated and comprehensive manner, that is, the responsibility must be shared between the control programs, TCP and HIV / AIDS.

The study population consisted of the PLWH in follow-up in these SCS that met the inclusion criteria: confirmed cases of HIV / AIDS, aged 18 years or more, residents in the municipality and not being inmates.

The calculation for the sample size was based on the survey of the total number of confirmed cases and monitored by the five clinics in January 2011, identifying a total of 1389 cases (249 belonging to the Clinic A, 374 to B; 249 to C, 374 to D, and 143 to E). The parameters considered were: the sample error of 5% (e = 0.05); 95% confidence interval (Z = 1.96) and P (population percentage) of 50%. It was obtained by the equation n_º_ = P. (1-P) Z^2^ / e^2^ a minimum sample size of 385 individuals. This value has been adjusted in relation to the total population (1389), resulting in 302 individuals.

The sampling process was performed in two stages: stratification with proportional sharing according to the SCS in charge of the case, and convenience sampling until the sample size was reached. Thus, taking into account the expected number of individuals in each SCS, 54 should be interviewed in SCS; 81 in B; 54 in C; 81 at D; and 31 in E. It is noteworthy that of the 302 individuals approached 49 refused to participate, resulting a total of 253 respondents. 

For data collection, interviews were carried out using a structured questionnaire developed from guidelines and protocols that address the organizational assessment of health care services for the care of HIV/AIDS^7^ ^-^ ^8^. This instrument was perfected using procedures related to content analysis, semantic analysis and pilot testing for readjustment and verification of its feasibility and the method suitable to meet the research objectives. Three professionals with expertise in the subject areas of HIV / AIDS, TB and public health were selected to perform the content analysis. They received an invitation letter, the data collection tool and the form to be examined. The semantic analysis was performed from the pilot test, in order to "*verify that all items are understandable to all members of the population to which the instrument is intended*"^15^. In the pilot test, 17 individuals were interviewed, following the pre-established inclusion criteria. It is emphasized that the subjects who participated in the pilot stage were not included in the final sample used in this study.

For this study, the selected variables related to the offering of health actions and services for the prevention of TB in PLWH were: proposal of blood tests (CD4 and viral load) requests for X-ray, offer of tuberculin test (TT), request of sputum smear (BK), provision of facial mask, supply of drugs to prevent the onset of opportunistic diseases, antiretroviral drugs, drugs for prevention of tuberculosis, questions about the existence of cough, fever, weight loss and loss of appetite in subjects and contacts, questions about the conditions of life, guidance on immune status, opportunistic infections, proper use of antiretroviral therapy (ART), signs and symptoms of TB, ways of TB transmission; care for the environment to avoid developing TB, care for the environment to prevent the spread of TB, reducing alcohol and drug use, food and nutrition; and request for government social benefits. The response categories of selected variables contemplated dichotomous scales, multiple choice and *Likert*, with values between "one" and "five" in which the most positive response corresponded to the highest value.

 Data collection was carried out between January 2012 and May 2013, in the SCSs, using rooms that ensured the privacy and anonymity of respondents. Data were analyzed using the software Statistica STATSOFT 9.0.

In the first stage of data analysis, in order to identify the supply of actions and services to PLWH by municipal SCS, indexes were created from the variables related to the actions and services. The indicator corresponded to the average value obtained by the sum of all the answers of all respondents to each question and divided by the total number of respondents^16^. Thus, each indicator was classified as unsatisfactory (between 1 and 2.5 corresponding to values up to 50% of the scale), intermediate (larger than 2.5 and smaller than 3.5 corresponding to values between 50% and 70% of the scale) or satisfactory (over 3.5 corresponding to values greater than 70% of the scale).

To classify the actions and services for the control of TB in PLWH, the measurement of the composite index was performed, corresponding to an average of the responses of all respondents for all selected variables.

According to the Resolution No. 196/96 of the National Health Council, this study had the approval of the Ethics Committee of the Ribeirão Preto School of Nursing, University of São Paulo, as per Protocol 1215/2010.

## Results

The actions and services for the control of TB in PLWH showed an average of 3.1 (SD = 1.87), and for that, the research participants classified them as intermediate.

**Figure 1:**
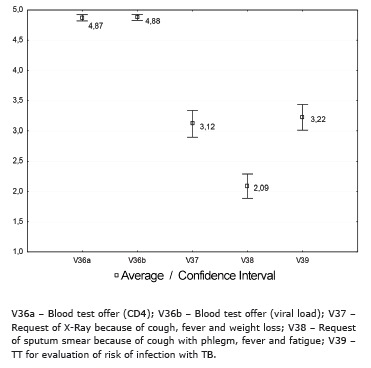
Average and confidence intervals distribution of the variables related to the offer of supplies, actions and health services by the reference team for controlling TB in people living with HIV/aids, Ribeirão Preto, SP, Brazil, 2012-2013

The indicators related to the offer for blood tests (CD4 and viral load) were the best evaluated and rated as satisfactory by respondents. However, the request of X-ray for respiratory signs, fever and weight loss was assessed as intermediate as well as the realization of TT for assessing the risk of TB infection. On the other hand, the request of BK because of the existence of cough with phlegm, fever and weight loss was considered unsatisfactory (Figure 1)

**Figure 2:**
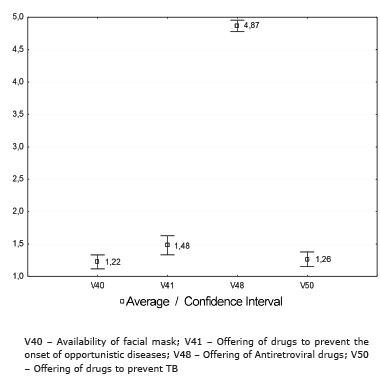
Average and confidence intervals distribution of the variables related to the offer of supplies, actions and health services by the reference team for controlling TB in people living with HIV/aids, Ribeirão Preto, SP, Brazil, 2012-2013

The indicators: availability of facial mask, offering of drugs to prevent the onset of opportunistic diseases and offering of drugs to prevent TB (Isoniazid) were considered unsatisfactory by PLWH (Figure 2).

**Figure 3:**
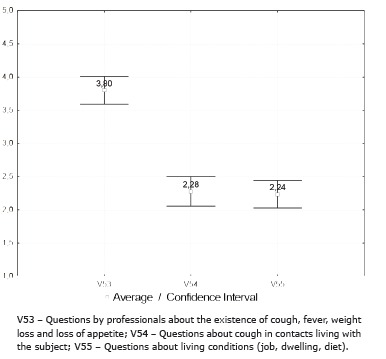
Average and confidence intervals distribution of the variables related to the questions about signs and symptoms suggestive of TB and offering of food and social incentives by the reference team for controlling TB in people living with HIV/aids, Ribeirão Preto, SP, Brazil, 2012-2013

As for the indicators related to testing for TB in PLWH, the quest by professionals about the existence of signs and symptoms suggestive of TB, cough, fever, weight loss and loss of appetite was classified as intermediate, and the indicator for questioning the existence of such signs and symptoms in contacts living with the subjects, obtained a unsatisfactory grade of performance (Figure 3).

**Figure 4:**
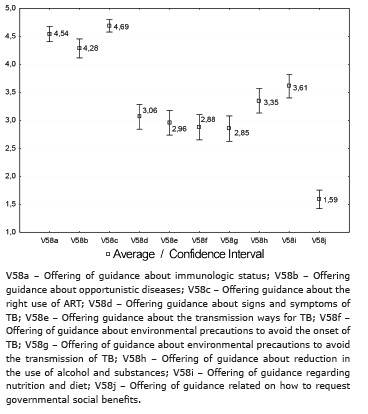
Average and confidence intervals distribution of the variables related to the offering of guidance by the reference team for controlling TB in people living with HIV/aids, Ribeirão Preto, SP, Brazil, 2012-2013

Regarding the provision of guidance by the reference team, most indicators were considered as intermediate, and it is important to emphasize that the guidelines related to TB fall within these indicators. However, offering information on immunologic status, opportunistic diseases and correct use of antiretroviral therapy, achieved satisfactory performance. The indicator related to the provision of guidelines for the request of government social benefits was found as unsatisfactory(Figure 4).

## Discussion 

The study identified the actions by the teams of SCS in the municipality geared towards the stabilization of HIV / AIDS on individuals, with a predominance of clinical and biological activities. In this regard, it is highlighted the satisfactory evaluation of respondents regarding the supply of blood tests (CD4 and viral load) and drugs for ART. This may relate to the consolidation of management strategies and control of AIDS, showing the programmatic commitment to maintain appropriate clinical management of the PLWH in follow-up^17^. It is essential to remark the important role played by representatives of Brazilian civil society in the achievement of public polices for assistance to HIV / AIDS, contributing significantly to the universal and free access to antiretroviral therapy, introduced in Brazil since 1996, which caused an impact relevant in mortality with increased survival from AIDS in the country^13^.

It is noteworthy that the regular use of antiretroviral therapy becomes an important protective factor against the development of TB in this population^18^. Furthermore, studies show that the use of antiretroviral therapy can prevent deaths among co-infected TB / HIV^19^ ^-^ ^21^, therefore, its use should be implemented as soon as possible in individuals diagnosed with HIV^7^. However, the isolated use of therapy does not guarantee the effective prevention of TB / HIV co-infection. It is also needed to think in a comprehensive way, considering the lifestyle, socioeconomic and cultural conditions as factors of vulnerability to contagion.

In this way, attention to PLWH should cover educational activities regarding prevention, mode of transmission, signs and symptoms and the importance of early diagnosis of TB, as well as teamwork strategies to seek suspect cases and early detection of TB cases in these subjects^8^.

With regard to diagnostic tests for TB (X-ray, TT and BK), the results show an intermediate and unsatisfactory supply despite the well-known vulnerability of PLWH for the developing of the disease. This low request may be related to the difficulties of individuals in recognizing the signs and symptoms of the disease and not reporting them during follow-up visits, and in the same way, health professionals may be disregarding the possibility of other comorbidities linked to HIV / AIDS, as TB.

Another aspect that can influence professional behavior is the lack of specific signs and symptoms of the disease in patients with HIV. In these cases, the diagnosis of TB is difficult as they present atypical radiographic findings, BK and negative sputum culture and a higher rate of extra pulmonary TB^22^.

Another reason for not requesting the tests concerns to the situation of the back-office of health services and laboratories to perform them. In Ribeirão Preto, during the data collection period, the X-ray machine was available in only one emergency room unit of the municipality, being the SCS C structure attached to this unit. That is, the "intermediate" request of this diagnostic test may have been influenced by this shortcoming.

A study conducted in three capital cities, Rio de Janeiro, Porto Alegre and Salvador, showed that low BK request was also a highlighted result(23), mainly because of the examination low cost and being at the same time being determinant for the diagnosis of TB pulmonary cases. Corroborating these findings, another study in Sao Jose do Rio Preto / SP found that this test is still not prioritized by health teams, despite being recommended as the primary method for the discovery of TB cases^24^.

Regarding the TT, it is well known that it is recommended by the MOH to be applied at least once a year in PLWH in order to investigate and get an early diagnosis of a possible TB infection^8^. However, in this study, it was observed that respondents rated the application of the TT as intermediate. Following this trend, WHO states that even with the widespread knowledge of the importance of interaction between the two endemics, only about 25% of PLWH treated in specialized health services have been subject to screening for TB in 2009^7^. The lack of trained professionals to take the reading in the municipality may be related to the low use of TT in the health services. It is worth noting that the professionals trained to perform this examination are part of the teams of the TCP, reinforcing the need for integration and coordination between both SCS and TCP programs.

The unsatisfactory offer of isoniazid for TB prevention may be related to the low performance of the TT by the SCS in the municipality, or may be related to the absence of infection. The TB treatment scheme is effective in about 95%, reducing the transmission of the disease. However, problems in treatment adherence, as the irregular use of medication and quitting treatment, can be identified as important factors affecting the effectiveness and, consequently, the control of tuberculosis in Brazil^25^.

The provision of drugs to prevent opportunistic infections was also rated unsatisfactory. Respondents only reported the use of trimethoprim-sulfamethoxazole association in a non-systematic way. It is important to remark that there is a supply of drugs to prevent opportunistic infections in the health care network, and the prescription was recorded as prescribed by the infectologists in the medical records. Thus, may be a bias in understanding by the respondent, confusing the use of these medications as part of the so-called "cocktail" that makes up the ARV treatment and not as related to the prevention of other situations referred to the disease. 

With regard to the questions posed to the PLWH looking for TB cases, it was identified an unsatisfactory and intermediate performance, which may show a poor use of a plan of care geared to PLWH. This situation may be related to the biologically oriented academic training, focused on a single disease / organ / system, to the absence or little evidence of completion of continuing education that emphasize new knowledge and recommendations, and also to a negligent look imposed on TB, even in a context of vulnerability to the disease as is the PLWH.

Thus, the continuous training of health professionals assumes a key role in the control of TB, particularly by the inclusion of training and continuous supervision in the practice of teams to identify gaps and difficulties in the process, honing the acquired skills^26^. Overcoming this obstacle also includes the training of the professional in the universities involving the integration teaching-service-research, as by including the guidelines of the TB control program, and enough time devoted to the theory and practice in order to cope with the disease and its prevention^11^.

With regard to the provision of guidance by the team, it is as well evident the importance given to issues related specifically to HIV / AIDS, and in this respect the guidelines were limited to clarification regarding the correct use of antiretroviral therapy, side effects and immunodeficiency resulting from the multiplication of HIV. With regard to guidance on TB, there is an intermediate offering of this action, which reiterates the neglect of the disease related to the care provided, involving here both technical and clinical issues as health information and education to the patient.

It is evident that the expert team has focused its actions in the clinical treatment, not exploiting enough the dimension of care in a wider level, possibly due to the increased workload in addition to the turnover of human resources, which precludes the updating of trained professionals.

Guidelines on diseases based on individual approaches constitute important elements for education and empowerment of the person to be able to perceive signs and symptoms of the disease, in the same way than to improve treatment adherence and strengthen the link between the professional and the individual^3^ ^,^ ^22^ ^,^ ^26^. Thus, to promote health education proposals able to educate the PLWH about the high likelihood of a TB infection, requires to extrapolate technical issues and, recurring to subjectivity and knowledge of them, sensitizing them with regard to transmission, care prevention, recognition of signs and symptoms and how to seek health services^24^ ^,^ ^27^ ^-^ ^28^.

Coupled with the social question, the supply of food and social benefits by the follow-up team was also classified as unsatisfactory by respondents. The social and economic dimensions are a challenge to be conquered and placed in the care plan. To clarify this point, only the SCS A, B and D had a social worker on the team, not available on a daily basis, nor offered to all individuals, just to those to whom the team judged relevant. At the time of data collection, the health unit housing the SCS C, had a social worker on staff, however, he was only responsible for the coverage of other specialties served in the clinic. This circumstance reflects, in addition to organizational aspects of the health system, issues related to the integration between professionals from different teams of the same service, as well as the integration of this team with teams of other services and / or specialties. This reinforces once again, the fragmentation of care and professional relationships. It is necessary to search for strategies that promote multidisciplinary and interdisciplinary work, using technologies centered on interpersonal relationships, allowing joint actions aimed at solving the demands and needs of PLWH^11^ ^-^ ^12^. 

Study limitations are the possibility of recall bias and selection bias due to the convenience sampling. Furthermore, the study only looked at the perception of the specific PLWH interviewed, which means that it may be permeated by personal and subjective components that involve satisfaction with the care provided.

## Conclusions

The offering of actions and services for the control of TB in PLWH in the municipality was considered intermediate, reinforcing the need for better planning of care for each individual. It is needed to adopt a stance that provides the connection between the different protagonists of care and to allows building a common care project by the team between TB and HIV / AIDS programs, consistent with the recognized needs, related to both conditions and available for access by professionals of all SCS and TCP.

The challenge that is posed implies the need to think in a comprehensive care, capable of articulating the offering of actions and health services for the control of TB in PLWH nested within each work process, with uniformity in the way of organizing assistance through the integration of professionals, teams and different health care network services.
